# Czech political candidate and donation datasets

**DOI:** 10.1038/s41597-025-04617-5

**Published:** 2025-02-19

**Authors:** Lukáš Linek, Michael Škvrňák, Michal Šoltés, Vítězslav Titl

**Affiliations:** 1 Institute of Sociology, Czech Academy of Sciences, Prague, Czech Republic; 2 Department of Economics and Empirical Legal Studies, Faculty of Law, Charles University, Prague, Czech Republic; 3 Utrecht University School of Economics, Utrecht University, Utrecht, Netherlands

**Keywords:** Politics, Government

## Abstract

This paper introduces a new Czech Political Candidate Dataset (CPCD), which compiles comprehensive data on all candidates who have run in any municipal, regional, national, and/or European Parliament election in the Czech Republic since 1993. For each candidate, the CPCD includes their first name, last name, age, gender, place of residence, university degree, party membership, party affiliation, ballot position, and election results for candidates and for parties. We match candidates over various elections by using algorithms that rely on their personal information. We add information on political donations made to political parties. We source donation information from the Czech Political Donation Dataset (CPDD), our other newly built dataset, in which we compile records of individual donations to 12 leading political parties from official records for the period from 2017 to 2023. CPDD is publicly available along with the CPCD.

## Background & Summary

The Czech Republic is a democratic unitary state structured into three levels of governance: municipalities (obce), regions (kraje), and the central government. Citizens thus participate in municipal, regional, and national parliamentary elections (the Czech parliament is bicameral, and the Chamber of Deputies and the Senate hold separate elections). With the introduction of European Parliament elections in 2004 and presidential elections in 2013, the frequency of elections has increased. The Senate and presidential elections operate on a two-round majority electoral system, while other elections employ a flexible list proportional representation system with large constituencies. In the flexible list system, voters influence the order of candidates on the party list by casting preference votes, effectively personalizing the elections, as votes for individual candidates impact seat distribution within parties. This institutional framework provides a rich context for studying political selection, candidate performance, multiple office holding, and political career trajectories across various elections and offices. The Czech Statistical Office (CZSO), which is in charge of processing all election results, facilitates such research by publishing official electoral results (including information about candidates stated on ballot lists) as open data immediately after each election. However, unlike, for example, Sweden, where candidates are assigned unique identifiers for traceability across elections^1^, the CZSO does not assign candidates such identifiers. Candidates thus cannot be easily tracked across years and types of elections. To address this issue and enhance research capabilities, we build a Czech Political Candidate Dataset (CPCD), which matches candidates across years and types of elections, consolidating data on candidates and district-level election results into a single, comprehensive dataset.

The CPCD offers candidate-level data that includes everyone who has run in any municipal, regional, national, and/or European Parliament election in the Czech Republic since the establishment of the independent state in 1993. The core variables provided by the CZSO include the candidate’s first and last names, age, place of residence, academic title, party affiliation, ballot position, and election outcomes for candidates and for parties. By employing an algorithm that integrates information on first and last names, year of birth, and place of residence, we merge individual candidates across different years and types of elections. This results in an unbalanced panel data structure in which candidates are observed multiple times, reflecting the number of elections they participated in. Overall, the dataset includes 841,565 unique candidates, and a total of 1,716,471 candidate-election observations. By providing this information, the CPCD aligns with similar datasets created for Norwegian^2^ and European Parliament elections^3^.

Additionally, we extract information on candidates’ gender and education level from the details provided on ballot lists. To identify candidates’ gender, we use name dictionaries and surname endings, and determine educational attainment using a dictionary of university degrees and academic titles. We further enhance the candidate data by linking it with data on donations made by individual candidates. This information is sourced from our newly developed Czech Political Donation Dataset (CPDD), in which we compile records of individual donations to political parties. We make this database publicly available along with the CPCD. We obtained the primary donation data, covering the period from 2017 to 2023, from the Office for Supervision of Economic Affairs of Political Parties and Political Movements (OSEAPPPM). After cleaning the data, we match donors to a party and political candidates using their full name and birth year information.

The data on Czech political candidates provided by CZSO have been extensively used in previous research. Scholars have focused on party nomination strategies^4^, partisan structure of candidates^5–7^, party switching among candidates^6^, the number of women among candidates and elected officials^8,9^, multiple office holders^10^, preference voting^11,12^, ballot-order effects^13,14^, uncontested elections^7,15,16^, and electoral^17^ and ruling coalition formation^18^. Another stream of research uses information on municipal candidates and analyses the effect of political donations on party nomination strategies in municipal elections^19^ and on the allocation of public procurement contracts^20,21^, the effect of political representation on public procurement^22^ and on budget allocation^23,24^, and the effect of political salaries on electoral competition and incumbency advantage^25^. Additionally, research into democratic accountability link uses candidate data from national elections and matches them with information on MP’s parliamentary activities (oral questions, bill sponsorship, speeches, voting participation) and party discipline to investigate the links between parliamentary work, re-selection, and re-election^26–29^. The datasets we make publicly available should facilitate further electoral research and help academics to broaden their focus by (1) linking candidates across different elections and different types of elections, (2) providing transparent and verified variables for gender and educational attainment, and (3) matching political candidates and donors to political parties.

## Methods

This paper introduces two newly created datasets: the Czech Political Candidate Dataset (CPCD) and the Czech Political Donation Dataset (CPDD). We created the CPCD by processing and standardizing official electoral data provided by the CZSO. It combines primary datasets for each election: municipal, regional, national (Chamber of Deputies and the Senate), and European Parliament (presidential elections are not included). We then linked information about individual candidates across elections by matching the candidates.

The CPDD provides information on individual donations to the last decade’s 12 most prominent political parties. We downloaded the primary datasets from the Office for Supervision of Economic Affairs of Political Parties and Political Movements (OSEAPPPM), hand-cleaned, and merged them across years. The available data covers the period from 2017, when the OSEAPPPM was established, to 2023. We then matched this donation dataset to the CPCD using donors’ names and birth years.

Because the preparation of datasets requires work with personal information including full names, birth dates, place of residence, and financial donations, we requested an ethics evaluation of this project. This study was approved by the Research Ethics Committee of the Faculty of Social Sciences, Charles University (Approval No. 135).

In this section, we first provide institutional background for each election type to clarify the details of Czech elected offices and electoral systems. Then, we describe our acquisition of primary data sources, standardization of variables, and how we matched them across elections (candidates) and years (donations). Finally, we describe the linking of candidates with the donations dataset.

### Institutional background

The Czech Republic operates under a parliamentary system with a bicameral legislature consisting of the Chamber of Deputies and the Senate. The Chamber of Deputies has greater political power. It decides on the government through votes of confidence, it approves the annual state budget, and a majority of MPs can outvote a Senate and/or Presidential veto on bills.

The Czech Republic has two levels of territorial self-governance: municipalities and regions. On January 1, 2024, there were 6,254 municipalities, with an average population of 1,743 residents per municipality with the median being 452^30^. Municipalities are primarily responsible for local development and spatial planning, municipal policing, water supply and sewage management, waste management, public transportation, preschools, primary and lower secondary education, and social services. This structure grants a medium degree of local autonomy compared to other democratic countries, particularly in Europe^31^. Large cities can be organized into municipal districts, each with its own autonomy, elected bodies, and administration. As of the 2022 municipal elections, eight cities have adopted this structure, forming 140 sub-municipal units. The municipal assembly (zastupitelstvo obce) is responsible for electing a mayor (starosta obce) and a municipal committee (rada obce, similar to aldermen in some European countries) through a majority vote. However, a municipal committee is not established if the municipal assembly has fewer than 15 members. The same rules apply to municipal districts. In the capital city of Prague, elections are organized according to the municipal election rules.

The regional level of governance was introduced in 2000, when the first elections to regional assemblies were held. Regional elections are held in 13 regions. The regional level of governance is responsible for regional development, road networks, transport, tourism, health care, upper-secondary education, and environmental protection. Regional governments are politically weak and have little financial autonomy. This translates into a low level on the Regional Authority Index^32^. Regional assemblies (krajské zastupitelstvo) elect governors (hejtman kraje) and regional committees (rada kraje) by majority vote.

Elections to municipalities, regions, the Chamber of Deputies, and the European Parliament follow almost identical electoral rules: a flexible list proportional representation (PR) system with similar electoral formulae, thresholds, and large constituencies. Only Senate elections are held under a two-round majority electoral system. Table 1 summarizes the basic features of electoral systems. Nomination rules are also broadly similar across elections. Because Czech democracy is primarily constructed as a party democracy, only registered parties and their coalitions nominate candidates. Parties also determine the rank of their candidates on the ballot. It is possible for independent candidates to run in municipal and Senate elections. In municipal elections, independent candidates and their associations are registered after they submit a petition supporting their candidacy signed by 7 percent of registered voters (the requirements are lower for an individual candidate). In Senate elections, independent candidates are enrolled after they submit a petition supporting the candidate signed by 1,000 registered voters residing in the constituency.

**Table 1:** Features and Rules of Elections in the Czech Republic.

Municipal	Regional	Parliamentary	Parliamentary	European
**Elected assembly**				
6254 municipal assemblies 140 mun. districts assemblies	13 regional assemblies	Chamber of Deputies	Senate	Czech represent’s to European Parliament
**Seats**				
5 to 70^+^	45 to 65^+^	200	81	21 since ’14 22 in ’09 24 in ’04
**Electoral system**				
flexible PR	flexible PR	flexible PR	two-round majority	flexible PR
**Electoral formula**				
d’Hondt since ’02 Saint Laguë in ’94, ’98	d’Hondt (starting with 1.42)	d’Hondt since ’02 Hagenbach-Bischoff in ’96, ’98	.	d’Hondt
**Electoral threshold**				
5 percent	5 percent	5 percent	.	5 percent
**Nomination**				
political parties independent cand’s	political parties	political parties	political parties independent cand’s	political parties
**Preference voting rules**				
number of pref. votes equals to the number of seats	4 pref. votes; 5% thr (since ’12) 4 pref. votes; 10% thr (till ’08)	4 pref. votes; 5 % thr (since ’10) 2 pref. votes; 7% thr (’02, ’06) 4 pref votes; 10% thr (’96, ’98)	.	2 pref. votes; 5% thr

*Notes*: ^+^ based on population size and choice of previous council

In all elections held under the proportional representation system in the Czech Republic, party ranking and preference votes co-determine the final order of candidates on the electoral list. In all elections, preferential voting is voluntary. The number of preference votes and the threshold for jumping up the candidate list varies across elections and have changed over time (see Table 1 for more details). In municipal elections, the preference voting rules differ from other elections, as voters can support various candidates across different ballots. Voters have the same number of votes as the number of elected representatives and have three options for how to use their votes: (1.) They can choose one party and allocate all their votes to that party; in that case, each candidate receives 1 vote; (2.) They can tick individual candidates across the party lists; or (3.) They can combine the two options: tick candidates across party lists and then give the rest of the votes to their preferred party. In the latter case, the votes given to the party are distributed to candidates in sequence from the top of the list. Due to the mechanical redistribution of votes towards better-ranked candidates, the votes received cannot be interpreted as pure preference votes. The threshold for moving to the top of the list is set to 110% of the average number of votes for all candidates on the party list.

### Czech Political Candidate Dataset

To generate this dataset, we follow these steps: downloading primary datasets, creating candidate datasets for each election (election-specific datasets), transforming variables, and matching candidates across election types (election-type datasets) and across all elections (the final dataset).

For each election, we downloaded the primary datasets from the official website of the CZSO (https://volby.cz/opendata/opendata.htm). Data on the 1996 and 1998 Chamber of Deputies elections and the 1994 and 1998 municipal elections are not available as open source and were provided to us by the CZSO upon a formal request. The primary datasets include information on:

- characteristics of candidates derived from ballot lists;
- electoral results for each candidate and party at the constituency level;
- list of registered political parties and list of parties and electoral coalitions running in elections.

As a first step, we created election-specific datasets, which combine information from primary datasets for each election. The unit of observation is an individual candidate for whom we record (1.) variables common to all types of elections (e.g., candidates’ characteristics, party affiliation) and (2.) election-specific variables (e.g., constituency names and codes, party composition of the list for municipal elections). For each candidate, we record their first name, last name, age, place of residence, academic titles, party membership, party affiliation, ballot position, number of (preference) votes received, whether the candidate was elected, and the number of votes for the candidate’s party. In the process of building election-specific datasets, we created three new variables: *candidate*_*education*, *candidate*_*gender*, and *candidate*_*birthyear*. We describe all variables in the Data records section. We kept original variables from primary datasets for transparency while preparing the final dataset, so the CPCD contains both original and newly created variables.

After standardizing election-specific datasets, we merged files for the same type of elections over the years and created 6 election-type datasets: municipal districts, municipalities, regions, the Chamber of Deputies, the Senate, and the European Parliament. We then merged election-type datasets into the final dataset. To match candidates, we use deterministic matching that compares pairs of candidates from different elections based on their first name, last name, year of birth, place of residence, education, occupation, party membership, and nominating party. Candidates must pass a threshold to be matched, which is based on the similarity of records in the variables. Not all variables are treated equally; pairs of candidates with the same name are graded based on the correspondence in values, with first name, last name, and year of birth having the largest effect. In addition, first and last names are used as blocking variables, meaning that they must be equal for the candidate records to match. If two or more candidates share the same first and last name and the same year of birth, then candidates’ education, occupation, party membership, and nominating party are used. The correspondence of these variables is judged by equality, except for occupation, in which string distance is used. To adjust for seasonal variability of the election dates, the birth year—calculated as the difference between the election year and candidates’ reported age—is allowed to deviate by one year. The above-described method may result in under-matching candidates who have changed their last names, particularly female candidates who adopt new names after marriage. To assess the potential impact, we re-ran the matching algorithm for municipal elections without using candidates’ last names as a blocking variable. In effect, we match candidates only using their first name and birth year. Under this alternative matching, the average number of elections per candidate increased slightly, from 1.960 to 1.969. The effect was more pronounced among female candidates under the age of 50, for whom the average number of elections per candidate rose from 1.795 to 1.815–a difference of 0.02. The increase was smaller for the older female candidates, growing by just 0.004 from 1.561 to 1.565. This corresponds to the increase for male candidates. These findings suggest that while some female candidates may change their last names during an active political career in municipal elections, the phenomenon does not seem widespread.

The process of merging the files and matching the candidates was sequential as we matched candidates first within the election-type datasets and later in the final dataset. This approach allows us to maximize our utilization of the information on the place of residence; in municipal (and city district) elections, candidates can only run in the municipality in which they reside. We use the municipality as a blocking variable to ensure that candidates running in a city district belonging to a municipality were matched to candidates running in the same municipality. The downside of this approach is that a candidate who has moved to a different municipality is not recognized as the same individual. In all other types of elections, candidates can run in any constituency regardless of where they live, so place of residence is not used as a blocking variable for matching candidates in these elections, and candidates who have moved are matched.

### Czech Political Donation Dataset

Czech political party financing is built around direct state funding as the primary funding source for political parties, with private donations playing a moderate role and membership fees being relatively insignificant. State subsidies are provided based on national election results and the number of seats a party holds in the Chamber of Deputies, the Senate, and regional assemblies^33^. The dominant role of state funding is complemented by a liberal regime of private funds. While political parties are prohibited from accepting contributions from foreign sources, anonymous donors, and government-owned corporations, other donors (corporations and individual donors) were not restricted in terms of donation amounts until 2016. Since then, the cap on donations from both individual corporations and individual citizens has been set to CZK 3 million (EUR 120k) per year. Aside from general rules set forth by civil and tax codes, donations to independent candidates are not subject to specific regulations.

Individuals are allowed to make financial or non-financial donations to political parties (examples of non-financial donations include offering space for advertising banners, distributing flyers, or other types of volunteering in campaigns). Political parties must disclose a list of all donors annually, including additional individual information. Information about membership fees does not have to be disclosed unless the amount exceeds CZK 50,000 (EUR 2k) per year. At this point, a membership fee is considered a donation by law. Since 2017, a list of donors must be submitted to the Office for Supervision of Economic Affairs of Political Parties and Political Movements (OSEAPPPM) as part of annual financial reports. The OSEAPPPM is responsible for reviewing these reports and may request corrective action if inaccuracies or errors are identified, and may impose fines. However, the office lacks the means to verify the accuracy of the information provided, including information about individual donors.

We downloaded the primary data about political donations from individual donors from the OSEAPPPM https://udh.gov.cz/vyrocni-financni-zpravy-stran-a-hnuti), hand-cleaned it, and collapsed it for individual donors by year and donation type (financial and non-financial). We rely on full name, date of birth, and political party to merge the individual donors over the years. The dataset covers individual donations made between 2017 and 2023 to any political party that received at least 1.5% of votes in the 2017 or 2021 Chamber of Deputies elections. The list includes 9 parliamentary and 3 non-parliamentary parties. Among the covered years and political parties, only 9 donor lists (from the total of 78 year-political party pairs) are not provided in CSV files. For these year-political party pairs, we downloaded the PDF and extracted the information using OCR tools to digitally readable formats and then cleaned them in the same manner as the initially digitally readable formats. Our prepared deposit contains the original PDF files, intermediate CSV files, script, and final datasets for these cases.

The hand-cleaning process consists of correcting typos in names, adding missing diacritics, capitalizing the first letters, and swapping the first name and last name in cases where the typos are highly probable; if one of the names is a typical Czech first name, while the other is a typical last name.

Furthermore, we corrected the birthdates of donors. If two or more donors with the same name made a donation to the same political party, but their birthdates differ in some suspect manner, we tagged the birthdate as a typo and hand-corrected it. The three specific types of suspected typos we consider are the following. First, if the month and day were flipped (e.g., if two donors with the same names had a date of birth on July 6, 1985 (2 donations) and June 7, 1985 (1 donation), we tagged the latter as a typo and merged the donors into one with a July 6 date of birth). Second, a possible typo that changes the date of birth by exactly one digit (e.g., if two donors with the same names had a birthdate of July 6, 1985 (2 donations) and June 6, 1985 (1 donation), we tagged the latter as a typo and merged the donors into one with a birthdate of July 6). We refrained from editing for both types of typos when both versions of donors’ personal information were represented the same number of times, e.g., July 6, 1985 (1 observation) and June 6, 1985 (1 observation). Third, if the year of birth reported corresponds to the year of the donation (e.g., if two donors with the same names had a birthdate of July 6, 1985, and July 6, 2021, we corrected the latter to July 6, 1985).

From the total 78-year list of financial donations to political parties, 69 are provided in digitally readable files. These datasets contain 85,378 recorded donations (donors could make more than one donation yearly). Among those, the manual changes edited 1,253 (1.5%) last names, 1,257 (1.5%) first names, and 475 (0.6%) birthdates. Among non-financial donations, the data provided in digitally readable files contain 31,642 reported donations, and the manual edit changed 176 (0.6%) last names, 215 (0.7%) first names, and 81 birth dates (0.3%). Manual edits in datasets extracted from PDF files were more frequent, but combined edits that were triggered by typos and inaccuracies in the primary dataset and from extracting the dataset from PDF files.

Compared to a previously used dataset of Czech political donations^19^, CPDD covers more political parties but spans fewer years of donations. This difference arises from our deliberate choice to prioritize the use of official data submitted to the OSEAPPPM, ensuring the highest possible level of transparency. The earlier dataset was manually transcribed from physical printed documents stored in the Chamber of Deputies archives, making it difficult to replicate and verify. In contrast, the CPDD is constructed from an online, publicly accessible primary data source, allowing for easier verification and transparent replication. While this approach enhances the dataset’s credibility, it inevitably results in shorter historical coverage.

### Matching the Czech Political Candidate Dataset and the Czech Political Donation Dataset

Individuals (candidates and donors) who are represented in both CPCD and CPDD have the same *person*_*id* in both datasets. We first collapsed the CPCD to the candidate level and the CPDD to the donor level. We then matched the collapsed CPCD and the CPDD based on similarity between individuals’ first and last name, year of birth, and party. The matching links unique donor ID (*donor*_*id*, defined by a unique combination of the first and last name and the year of birth) with *person*_*id* in the candidate dataset. For all donors with a counterpart in the candidate dataset, we use *person*_*id* from the candidate dataset that links the two datasets. We created a new unique ID for donors that were not matched to candidates. This approach ensures that if a candidate and a donor are identified as one individual, the observations share the same *person*_*id*.

## Data Records

The CPCD and CPDD are publicly available at the Czech Social Science Data Archive (10.14473/CSDA/MDZC3F)^34^. The datasets are available in .csv and .rds formats. The script to produce final datasets, which covers the whole process from data download to matching CPCD and CPDD is available at OSF (10.17605/OSF.IO/RAKJZ)^35^.

### Czech Political Candidate Dataset

The deposit contains 6 election-type datasets and the final CPCD, which merges all candidates across elections. The CPCD contains information on 841,565 unique candidates and 1,716,471 candidate-election observations.

Table 2 provides statistics on the number of candidates for each election. The largest number of candidates participate in municipal elections, with an average of over 180,000 per election year. In contrast, fewer than a thousand candidates run in each Senate and European Parliament election. Several thousand candidates compete in elections for the Chamber of Deputies and in regional elections. For each election, the table further provides the number of elected representatives, the share of elected among all candidates, the number of female candidates, the number of elected female candidates, and finally, the share of female candidates among elected candidates.

**Table 2:** Number of Candidates.

Election	Number of Candidates	Elected Candidates	Share of Elected Candidates	Female Candidates	Elected Female Candidates	Share of Female Among Elected (%)
**Municipal**						
1994	145,611	59754	41.0	31,530	10,614	17.8
1998	163,664	59986	36.7	40,784	12,263	20.4
2002	177,309	60001	33.8	48,098	13,567	22.6
2006	186,716	60056	32.2	54,988	15,000	25.0
2010	208,510	59793	28.7	65,064	15,725	26.3
2014	211,295	59573	28.2	68,091	16,129	27.1
2018	195,630	59331	30.3	64,249	16,606	28.0
2022	178,432	59228	33.2	59,459	17,163	29.0
**City districts**						
1994	13,967	2406	17.2	4,322	527	21.9
1998	15,945	2426	15.2	5,477	528	21.8
2002	15,621	2493	16.0	5,434	615	24.7
2006	14,948	2519	16.9	5,421	661	26.2
2010	17,923	2545	14.2	6,585	711	27.9
2014	22,197	2548	11.5	8,206	730	28.6
2018	20,531	2561	12.5	7,828	729	28.5
2022	16,791	2552	15.2	6,648	756	29.6
**Regional**						
2000	7,725	675	8.7	1,684	97	14.4
2004	8,309	675	8.1	2,164	103	15.3
2008	8,206	675	8.2	2,395	119	17.6
2012	11,304	675	6.0	3,118	134	19.9
2016	11,803	675	5.7	3,545	137	20.3
2020	9,711	675	7.0	2,671	149	22.1
**Chamber of Deputies**						
1996	3,909	200	5.1	789	30	15.0
1998	3,374	200	5.9	704	30	15.0
2002	6,068	200	3.3	1,597	34	17.0
2006	4,985	200	4.0	1,385	31	15.5
2010	5,022	200	4.0	1,366	44	22.0
2013	5,899	200	3.4	1,588	39	19.5
2017	7,524	200	2.7	2,154	44	22.0
2021	5,242	200	3.8	1,658	50	25.0
**Senate**						
1996	568	81	14.3	59	9	11.1
1998	137	27	19.7	12	3	11.1
2000	160	27	16.9	26	4	14.8
2002	168	27	16.1	26	3	11.1
2004	197	27	13.7	37	3	11.1
2006	204	27	13.2	39	6	22.2
2008	200	27	13.5	34	5	18.5
2010	227	27	11.9	37	4	14.8
2012	233	27	11.6	42	5	18.5
2014	242	27	11.2	37	5	18.5
2016	233	27	11.6	43	6	22.2
2018	236	27	11.4	42	2	7.4
2020	235	27	11.5	38	4	14.8
2022	178	27	15.2	43	9	33.3
By-elections	125	15	12.0	24	2	13.3
**European Parliament**						
2004	809	24	3.0	205	5	20.8
2009	731	22	3.0	210	4	18.2
2014	857	21	2.5	230	5	23.8
2019	841	21	2.5	201	7	33.3
2024	674	21	3.1	245	8	38.1

*Notes*: For each election covered in the dataset, this table shows the number of candidates who ran (valid candidates only, col. 2), the number of elected candidates (col. 3), the share of elected candidates from those who ran (col. 4), the number of female candidates who ran (col. 5), the number of female elected candidates (col. 6), and the share of female among elected candidates (col. 7).

The CPCD records 31 variables common for all elections and additional 13 election-specific variables. We describe the common variables here and refer the readers to the Codebook (see Supplementary Information A) for a description of election-specific variables. Table 3 lists the original names of the common variables retrieved from primary datasets, the CPCD names, and includes labels that describe the variable. Variables can be broadly grouped into three categories: election, candidate, and election results. The first category contains *election*_*year*, *election*_*date* (first day of the election), and *election*_*type*.

**Table 3:** Czech Political Candidate Dataset: Description of Variables.

Original Variable Name	CPCD Variable Name	CPCD Variable Label
**Election**		
N/A	election_year	Election: year
DATUMVOLEB, N/A	election_date	Election: date
N/A	election_type	Election: type
**Candidate**		
N/A	person_id	Candidate: identification code
JMENO	candidate_name	Candidate: name
PRIJMENI	candidate_surname	Candidate: last name
VEK	candidate_age	Candidate: age
N/A	candidate_birthyear	Candidate: year of birth
N/A	candidate_gender	Candidate: gender
TITULPRED	candidate_title_before	Candidate: self-reported academic titles before name
TITULY	candidate_title_both	Candidate: self-reported academic titles before and after name
TITULZA	candidate_title_after	Candidate: self-reported academic titles after name
N/A	candidate_education	Candidate: education in 6 categories (self-reported)
POVOLANI	candidate_occupation	Candidate: occupation
BYDLISTEK	candidate_place_code	Candidate: place of living - code
BYDLISTEN	candidate_place_name	Candidate: place of living - name
KSTRANA	candidate_partyrun_code	Candidate: party running the election - code
ZKRATKAK8	candidate_partyrun_name	Candidate: party running the election - abr. name
NAZEV_STRK	candidate_partyrun_fullname	Candidate: party runnig the election - full name
NSTRANA	candidate_partynom_code	Candidate: nominating party - code
ZKRATKAN8	candidate_partynom_name	Candidate: nominating party - abr. name
PSTRANA	candidate_partymem_code	Candidate: party membership - code
ZKRATKAP8	candidate_partynmem_name	Candidate: party membership - abr. name
PORCISLO	candidate_ranking	Candidate: ranking on the list
PLATNOST	candidate_validity	Candidate: validity of candidacy
**Election results**		
POCHLASU	candidate_voteN	Candidate: number of preference votes
POCPROCVSE	candidate_voteP	Candidate: percent of preference votes
HLASY_K1	candidate_voteN_SR1	Candidate: number of votes (Senate 1)
HLASY_K2	candidate_voteN_SR2	Candidate: number of votes (Senate 2)
URIZ_PR_K1	candidate_voteP_SR1	Candidate: percent of votes (Senate 1)
URIZ_PR_K2	candidate_voteP_SR2	Candidate: percent of votes (Senate 2)
MANDAT	seat	Candidate: seat - yes/no
PORADIMAND	ranking_seat	Candidate: ranking for seat distribution (after election ranking)
PORADINAHR	ranking_subs	Candidate: ranking of substitutes (after election ranking)
HLASY	party_voteN	Party: number of votes in constituency
PROCHLASU	party_voteP	Party: percent of votes in constituency

*Notes*: Variables *candidate*_*voteN* and *candidate*_*voteP* are recorded only for flexible PR systems; for Senate elections, these variables have different suffixes (*candidate*_*voteN*_*SR*1, *candidate*_*voteN*_*SR*2, *candidate*_*voteP*_*SR*1, *candidate*_*voteP*_*SR*2).

The second and largest category of variables includes a candidate’s ID (*person*_*id*) and pre-election information, i.e., information voters would know (or could easily infer) before voting. The former is a unique identifier for each candidate created during the merging process, with a structure that reflects the number and types of elections in which a candidate ran. The latter largely adopt variables from the primary datasets, including first name, last name, age, academic titles, place of residence (name and code), ballot position, and party membership and affiliation. We also keep occupation as a string variable in the Czech language.

We created three new variables: birth year, gender, and education. Variable *candidate*_*birthyear* was created as the difference between the election year and the age of a candidate announced on the ballot lists. Variable *candidate*_*gender* classifies candidates as male or female based on the dictionary of male and female first names from the Ministry of the Interior. If both males and females use the same first name, we identify females based on the ending of their last name and the name of the occupation provided by candidates on the ballot list, as Czech women’s last names and words for occupations have predominantly gender-specific endings. The variable *candidate*_*education* builds on academic titles stated on the ballot list. We use a dictionary of academic titles to categorize candidates into six education levels: (1.) no university education; (2.) BA degree; (3.) MA degree; (4.) PhD degree; (5.) associate professor (docent, habilitation); (6.) full professors. Similar categorization has been used in previous research^11^. Two notes about academic titles are worth mentioning. First, academic titles are self-reported with no formal validation process. Second, Czech voters seem to interpret academic titles (especially medical and law titles) as a sign of a candidate’s expertise and tend to cast more votes for educated candidates, further reinforcing candidates’ interest in stating their academic titles^11,19^.

The final dataset also contains three variables regarding candidates’ party membership and party affiliation, which come directly from primary datasets. The variable *candidate*_*partymem* refers to formal party membership. The variable *candidate*_*partyrun* provides the name of the list of candidates running the election. If a single party forms a ticket to run in elections, this variable is the same as the name of the party. However, when parties form a coalition, the name of the list differs from that of allied parties and reflects the nature of a coalition. Sikk and Köker (2019) refer to this variable as *electon*^36^. Finally, the variable *candidate*_*partynom* signals which party nominated a candidate and corresponds to the endorsing party in the coalition. The dataset contains an abbreviated party name (acronym) and the party’s numerical code for all three party-related variables. The codes come from the list of registered political parties provided by the Ministry of the Interior, which contains long names, short names, and numerical codes for each party. The name and numerical code are identical across all elections if the party is organizationally stable. We provide an unabbreviated name only for *candidate*_*partyrun*. If independent candidates run in municipal and Senate elections, then the variables *candidate*_*partyrun* and *candidate*_*partynom* record a code “independent candidate”. The dataset also contains information on where a candidate lives (*candidate*_*place*_*code*, *candidate*_*place*_*name*). In the case of municipal elections, this variable also shows the city or village where a municipal assembly was elected.

Finally, the variable *candidate*_*validity* captures whether a candidate actually ran in an election. Because candidate lists are submitted almost two months before an election, candidates may die, withdraw their candidacy, or be dismissed by the party before the election. Their ballot rank (*cand*_*ranking*) is recorded as if they ran for election. However, preference votes are recorded as 0 or N/A. If we restrict the data to candidates who actually ran in elections, the number of unique candidates drops from 841,565 to 838,656 and the number of candidate-election observations falls from 1,716,471 to 1,708,049.

The third category contains post-election variables, i.e., variables regarding the election results. This includes the preference votes received in absolute values (*candidate*_*voteN*) and as a percentage of all preference votes given to a candidate’s party (*candidate*_*voteP*). In municipal elections, *candidate*_*voteP* is given as a percentage of all votes a candidate’s party list received. After preference votes are considered, the candidate’s final ranking is provided (*ranking*_*seat*, *ranking*_*subs*). Finally, we add the absolute and relative number of votes a candidate’s party received in the constituency (*party*_*voteN*, *party*_*voteP*).

The Senate election data differ slightly, as the two-round majority system tends to lead to two election rounds. For both rounds, we record the absolute and relative number of votes for candidates and whether a candidate was elected. Two variables, *candidate*_*voteN* and *candidate*_*voteP*, are recorded only for flexible proportional representation systems (municipal, regional, Chamber of Deputies, European Parliament), while for the Senate we provide four different variables with the same root name, but different suffixes (*candidate*_*voteN*_*SR*1, *candidate*_*voteN*_*SR*2, *candidate*_*voteP*_*SR*1, *candidate*_*voteP*_*SR*2).

Additionally, the dataset contains variables related to the constituency in which the election took place. For municipal elections, this includes the municipality ID (*municipality*_*id*), municipality name (*municipality*_*name*), and the city district ID (*city*_*district*_*id*). The municipality ID remains the same over time. If a part of a municipality detaches from its original municipality, the newly formed municipality is assigned a new ID, while the original municipality retains its existing ID. For regional elections, the dataset contains the region ID (*region*_*id*) and name (*region*_*name*). Finally, the constituency for Senate elections is captured by the district number (*senate*_*const*_*id*), while for the Chamber of Deputies, we use *chamber*_*const*_*id*. Note that the electoral region number changed in 2002 due to electoral reform.

### Czech Political Donation Dataset

The final dataset contains 57,339 donor-party-year unit observations and 38,472 unique donors. Tables 4 and 5 report the number of unique donors and the total sum of financial and non-financial donations by political party and year, respectively. It also indicates which data is from digitally readable sources and which are extracted from PDF files. For each observation, the dataset records the political party to which the donation was addressed (*donation*_*party*), the first name (*donor*_*name*), last name (*donor*_*surname*), year of birth (*donor*_*birthyear*), year of donation (*donation*_*year*), amount of total donation (*donation*_*all*), amounts of financial (*donation*_*financial*) and non-financial (*donation*_*nonfinancial*) donations, ID (*donor*_*id*) indicating unique donors and person ID (*person*_*id*) that links the CPDD and the CPCD. Finally, the dataset also includes information on whether the observation is from digitally readable documents or extracted from PDF files (*donation*_*source*). The list of variables is presented in Table 6. Matching the CPDD with the CPCD leads to 18,594 matches, indicating that 32% of the number of donations (and 53% of donations value) were made by candidates.

**Table 4:** Number of Unique Donors.

	2017	2018	2019	2020	2021	2022	2023
**KDU-ČSL**	1,759	778	1,790	2,080	^+^559	486	280
**KSČM**	275	240	214	228	117	54	36
**ODS**	768	1,299	469	1,101	584	1,155	383
**Piráti**	2,207	531	929	1,640	2,511	1,329	779
**SPD**	1,114	^+^188	^+^184	^+^157	^+^83	^+^267	^+^48
**STAN**	386	331	71	947	250	559	155
**TOP 09**	802	559	582	583	100	456	95
**ANO**	764	1,034	737	^*^828	^+^623	746	845
**ČSSD**	129	241	37	155	55	79	14
**Svobodní**	1,257	943	329	247	342	400	751
**Trikolora**	.	.	6,478	3,772	^+^2,015	667	422
**Přísaha**	.	.	.	.	714	121	43

*Notes*: This table shows the number of unique donors by political party and year. “.” stands for years before the political party was established. ^+^ and ^*^ indicate that the primary data were extracted from PDF documents, with the latter being more prone to flawed extraction of information.

**Table 5:** Value of Donations (in Mil. CZK).

	2017	2018	2019	2020	2021	2022	2023
**KDU-ČSL**	23.9	12.9	16.1	18.5	^+^8.9	15.1	7.2
**KSČM**	9.2	8.3	7.0	6.8	3.9	1.5	0.5
**ODS**	34.7	51.7	11.4	40.4	16.7	50.1	11.6
**Piráti**	4.5	3.3	2.3	5.4	10.2	8.5	4.3
**SPD**	5.0	^+^2.5	^+^2.5	^+^1.6	^+^4.1	^+^2.6	^+^0.8
**STAN**	13.6	6.5	3.2	11.3	4.8	17.6	5.9
**TOP 09**	10.6	16.9	6.8	8.8	3.9	16.8	2.9
**ANO**	22.8	36.2	12.0	^*^21.3	^+^12.1	27.0	11.8
**ČSSD**	4.0	10.7	1.8	9.5	3.1	3.8	3.3
**Svobodní**	2.5	2.7	1.1	0.6	2.4	1.3	1.3
**Trikolora**	.	.	6.7	12.8	^+^14.5	2.3	0.8
**Přísaha**	.	.	.	.	5.8	2.1	1.7

*Notes*: This table shows the aggregate value of financial and non-financial donations in mil CZK by political party and year. “.” stands for years before the political party was established. ^+^ and ^*^ indicate that the primary data were extracted from PDF documents, with the latter being more prone to flawed extraction of information.

**Table 6:** Czech Political Donation Dataset: Description of Variables.

CPDD Variable Name	CPDD Variable Label
donor_id	Donor: identification code
person_id	Person identification code (identical to *person*_*id* in CPCD)
donation_party	Political party to which the donation was addressed
donation_year	Year of donation
donor_name	Donor: first name
donor_surname	Donor: surname
donor_birthyear	Donor: year of birth
donation_all	Total value of individual financial and non-financial donation in a given year
donation_financial	Total value of individual financial donation in a given year
donation_nonfinancial	Total value of individual non-financial donation in a given year
donation_source	From digitally readable document = 1; extracted from PDF = 2

*Notes*: This table lists the variables in the CPDD. For donors who are also a political candidate, the personal ID is the same in CPDD and CPCD.

## Technical Validation

This section provides technical validation for both the CPCD and the CPDD datasets. We developed several procedures to ensure data quality and reproducibility of the results. The aim of this section is to describe the logic of the validation and its results. This quality assurance process is largely implemented in R.

### Technical validation for CPCD

We implemented three technical validity checks for the CPCD. First, we ensured that the variables contain valid codes, names, and numerical values. Specifically, we verified that the percentage of preference votes fell between 0 and 100, the absolute number of votes was non-negative, and that other variables, including gender, education, age, seat, and birth year, were free of missing or invalid data. We also checked whether the CPCD recorded 0 votes for candidates who eventually did not run in an election. Finally, we compared the sum of the variable *seat*, an indicator for elected status, to the number of seats filled for each election and each constituency. Counts based on the CPCD shown in Table 2 match exactly the numbers provided by the CZSO.

Second, we compare the CPCD to two publicly available datasets that partly cover Czech political candidates: Comprehensive European Parliament electoral data (COMEPELDA) and the dataset used in the Party People book that studies candidate turnover in elections in Central and Eastern Europe^37^. We also intended to compare the CPCD to the Constituency-Level Elections Archive (CLEA), but abandoned the plan after we found that the CLEA dataset was flawed and inconsistent with the primary and the only authoritative dataset provided by the CZSO.

COMEPELDA consolidates information on European Parliament elections into one source. It provides information on formal electoral rules as well as national-level and district-level election results for parties and individual politicians (including full candidate lists)^3^. In the case of parties covered by COMEPELDA, the number of candidates running on their party lists is the same. In addition, the comparison of individual candidates, the number of preferential votes, and elected MEPs is the same in both datasets.

We utilize the dataset used in the Party People book to validate our matching of candidates across elections. Specifically, we replicate the Czech parties’ weighted candidate novelty (WCN) in two consecutive elections to the Chamber of Deputies for the period from 1998 to 2013 from Sikk and Köker^36^. Candidate novelty measures the share of candidates who did not run in any previous election, which is then weighted by the candidates’ list position and the parties’ vote share, to calculate WCN. Our measure of WCN strongly correlates (r > 0.9) with the WCN reported by Sikk and Köker^36^; see Tables S2–S6 in Supplementary Information B.

Third, we identified recently published academic articles on females among elected representatives in the Czech Republic^8,38^ and compared the numbers to those we derived using the CPCD. The variable indicating female candidates in the CPCD was created based on the names of the candidates (see section Data records), so the comparison of female shares provides further validation of the data transformations. We present the share of females among elected representatives for all elections in the last column of Table 2. The first study compared, by Maškarinec^8^, graphically presents the share of women among elected representatives for the Chamber of Deputies, the European Parliament, and regional and municipal assemblies. The graphical comparison of our statistics and the figure from Maškarinec^8^ suggests that there are no discrepancies in the numbers of female elected officials between his study and our data. The second study, by Voda (2022), provides exact numbers for seven municipal elections. We record the same numbers for three elections (2010, 2014, and 2018) and differ by 0.1 percentage points in three other elections (1994, 1998, and 2006). In the 2002 election, the difference was larger, as CPCD statistics yield 22.6% of female elected candidates, while the study by Voda (2022) says 27.1%. As CPCD’s 22.6% corresponds to the same figure presented in the study by Maškarinec^8^, we believe the CPCD yields more credible statistics.

### Technical validation for CPDD

To validate the CPDD dataset, we used donors’ dates of birth. Because public authorities lack the means to verify the accuracy and correctness of the submitted list of donors, there could be some concern that reported donors are made up. Personal donor information, such as date of birth, would be the primary suspect, as this piece of information is the most challenging to verify. We build on psychological literature that argues that people have difficulty generating random digits to test for potentially made-up dates of birth, similar to what is known in the literature as election forensics to detect fraud in election results^39,40^.

We test that day-in-month donors’ dates of birth are distributed with an equal frequency. We collapsed donors for a given party over the period studied, so that each donor is counted once regardless of the value or the number of donations made. Table 7 shows Pearson *χ*^2^ statistics, the corresponding p-value, and the most frequent day for each political party considered. The p-value is (weakly) larger than a 0.05 significance level for each political party. However, KSČM (p-value of 0.063) and Přísaha (p-value of 0.050) are on the margin of statistical significance. Figures S1, S2, and S3 in Supplementary Information C show the distance from the average number of donors born on a given day by a political party. Note that we restrict the sample to days between the 1^st^ and 28^th^, as subsequent days are predicted to be represented less in a random sample of dates. Similarly, the argument of random allocation of day-in-month dates does not generalize to months, as births are not randomly distributed over a year.

**Table 7:** Distribution of Day-in-Month among Donors’ Date of Birth.

	Pearson *χ*^2^ (27)	p-value	Most frequent day
**KDU-ČSL**	20.44	0.811	24^th^
**KSČM**	39.00	0.063	1^st^
**ODS**	28.53	0.384	14^th^
**Piráti**	30.59	0.288	21^st^
**SPD**	35.76	0.120	25^th^
**STAN**	17.13	0.928	16^th^
**TOP 09**	21.37	0.768	24^th^, 28^th^
**ANO**	24.10	0.625	2^nd^
**ČSSD**	30.85	0.277	2^nd^
**Svobodní**	32.76	0.205	4^th^
**Trikolora**	21.24	0.775	13^th^, 19^th^
**Přísaha**	40.11	0.050	20^th^
**All donors**	23.97	0.632	20^th^, 21^th^

*Notes*: This table shows results from a formal test of uniformly distributed day-in-month among donors by a political party. All p-values are (weakly) larger than 0.05. However, the test results for KSČM and Přísaha are on the margin of being statistically significant at a 0.05 significance level. The bottom row shows the most frequent birth day.

Compared to other information provided in the dataset, dates of births are the least verifiable from public sources. Therefore, we believe that the lack of evidence of manipulating birth dates is promising evidence that the other information was not manipulated either.

## Supplementary information

Supplement information

## Data Availability

The CPCD and CPDD are publicly available: 10.14473/CSDA/MDZC3F^34^. The code to replicate the construction of the final datasets introduced in this paper and the analysis presented is available through the OSF repository https://osf.io/rakjz/^35^. All files use UTF-8 encoding.
