# Supplementary material for: Czech political candidate and donation datasets
Source: Sci Data. 2025 Feb 19;12:302. doi: 10.1038/s41597-025-04617-5 (PMC11840027; doi:10.1038/s41597-025-04617-5)
Supplement: Supplementary file 1 — Supplement information [file 41597_2025_4617_MOESM1_ESM.pdf]

Czech political candidate and donation datasets: Supplementary information

This document presents supplementary materials related to the Czech political candidate and donation datasets. There are three sections within the Supplementary Information:

Supplementary Information A: Codebook for CPCD
Supplementary Information B: Results of the validation tests
Supplementary Information C: Distribution of day-in-month donors' dates of birth

Supplementary Information A: Codebook for CPCD

The codebook lists all variables included in the final Czech Political Candidate Dataset (CPCD). For each variable, the codebook includes the original name of the variable from the primary datasets provided by the Czech Statistical Office (CZSO), the CPCD name of the variables, a description of the variable, and a note.

Table S1. Codebook

CPCD Variable Name	Original Variable Name	CPCD Variable Label	Note
election_year		Election: year	
election_date	DATUMVOLEB, N/A	Election: date	First day of election; except for the 2000 regional elections, all went for two days.
election_type		Election: type	Nominal: municipal, mun. district, regional, Chamber of Deputies, Senate, European Parliament
person_id		Candidate: identification code	
candidate_name	JMENO	Candidate: name	
candidate_surname	PRIJMENI	Candidate: last name	
candidate_age	VEK	Candidate: age	
candidate_birthyear		Candidate: year of birth	
candidate_gender		Candidate: gender	Nominal: male, female
candidate_title_before	TITULPRED	Candidate: self-reported academic titles before name	
candidate_title_after	TITULZA	Candidate: self-reported academic titles after name	
candidate_title_both	TITULY	Candidate: self-reported academic titles	
candidate_education		Candidate: education in 6 categories (self-reported)	Nominal: no title, Bachelor, Master, Doctor, Associate Professor (docent), Professor
candidate_occupation	POVOLANI	Candidate: occupation	String: in Czech language; not available for 1994 municipal election
candidate_place_code	BYDLISTEK	Candidate: place of living - code	Numerical
candidate_place_name	BYDLISTEN	Candidate: place of living - name	String: in Czech language
candidate_partyrun_code	KSTRANA	Candidate: party running the election - code	Numerical: list running the election (kandidující strana), administrative ranking of parties, decided by a lottery; not for municipal and Sena
candidate_partyrun_name	ZKRATKAK8	Candidate: party running the election - abr. name	Nominal: list running the election (kandidující strana), abbreviated name; not for municipal and Senate elections
candidate_partyrun_fullname	NAZEV_STRK	Candidate: party running the election - full name	Nominal: list running the election (kandidující strana); full name
candidate_partynom_code	NSTRANA	Candidate: nominating party - code	Numerical: party nominating candidate (nominující strana); num. code; not available for Chamber of Deputies 1996, 1998
candidate_partynom_name	ZKRATKAN8	Candidate: nominating party - abr. name	Nominal: party nominating candidate (nominující strana); abbreviated name
candidate_partymem_code	PSTRANA	Candidate: party membership - code	Numerical: party membership of candidate; num. code
candidate_partynmem_name	ZKRATKAP8	Candidate: party membership - abr. name	Nominal: party membership of candidate; abbreviated name
candidate_ranking	PORCISLO	Candidate: ranking of candidates on the list	Numerical: not standardized; not for the Senate
candidate_validity	PLATNOST	Candidate: validity of candidacy	Dichotomic: candidate ran election: yes (0); no (1)
candidate_voteN	POCHLASU	Candidate: number of preference votes	Numerical; only for flexible list PR systems
candidate_voteN_SR1	HLASY_K1	Candidate: number of votes (Senate 1)	Numerical; only for the Senate
candidate_voteN_SR2	HLASY_K2	Candidate: number of votes (Senate 2)	Numerical; only for the Senate
candidate_voteP	POCPROCVSE	Candidate: percent of preference votes	Numerical; only for flexible list PR systems
candidate_voteP_SR1	URIZ_PR_K1	Candidate: percent of votes (Senate 1)	Numerical; only for the Senate
candidate_voteP_SR2	URIZ_PR_K2	Candidate: percent of votes (Senate 2)	Numerical; only for the Senate
seat	MANDAT	Candidate: seat - yes/no	Dichotomic: candidate did not win seat (0), candidate won seat (1)
ranking_seat	PORADIMAND	Candidate: ranking for seat distribution (after election)	Numeric; only for those candidates that won a seat; not available for Chamber of Deputies 1996, 1998 and 2002, and for municipal electio
ranking_subs	PORADINAHR	Candidate: ranking of substitutes (after election)	Numeric; only for those candidates that did not win a seat; not available for Chamber of Deputies 1996, 1998 and 2002, and for municipal
party_voteN	HLASY	Party: number of votes in constituency	Number of votes for a list in constituency. In mun. and mun. district elections, it sums candidate_voteN
party_voteP	PROCHLASU	Party: percent of votes in constituency	Numerical: percent of votes for a list in constituency
party_rank	POR_STR_HL	Party: election number	Numerical: administrative ranking of parties, decided by a lottery, only for municipal elections
senate_candidate_no	CKAND	Candidate: election number	Numerical: administrative ranking of candidates, decided by a lottery; only for the Senate
municipality_type	TYPZASTUP	Type of municipality	Dichotomic: city (1), city district (2), only for municipal elections
municipality_id	KODZASTUP	Municipality: ID	Numeric: code of municipality from state registers, only for municipal elections
municipality_name	KODZASTUP_NAZEV	Municipality: name	String: name of municipality from state registers, only for municipal elections
municipality_const_id	COBVODU	Municipality: constituencies	Numeric: for constituency in municipal elections
city_district_id		Municipality: city districts	Numeric: for city districts in municipal elections
region_name	KRAJ_NAZEV	Regional assembly: constituency - name	String: for constituency
region_id	KRZAST	Regional assembly: constituency - code	Numeric: for constituency
electoral_const_id	VOLKRAJ	Chamber of Deputies: constituency	Numeric: for constituency
senate_const_id	OBVOD	Senate: constituency	Numeric: for constituency
candidate_citizenship	STATOBCAN	Candidate: citizenship	Nominal; only for EP election

Supplementary Information B: Results of the validation tests

This appendix presents results from the technical validation. Tables S2, S3, S4, S5, and S6 compare the weighted candidate novelty measure calculated based on CPCD and the original dataset developed by Sikk and Köker.[1]

Table S2. Comparison CPCD and Party People dataset, Chamber of Deputies Election 1998
Weighted Candidate Novelty

Political Party	Sikk & Köker	CPCD
A2001	1.00	1.00
CAO	0.61	0.71
CSNS	0.43	0.45
CSSD	0.22	0.14
DEU	0.30	0.32
DZJ	0.45	0.38
KDU-CSL	0.18	0.17
KSCM	0.09	0.06
MODS	0.07	0.06
NEZ	0.79	0.82
ODA	0.57	0.26
ODS	0.48	0.47
OK	0.83	0.88
PB	0.32	0.31
SDCR	0.86	0.94
SPR-RSC	0.16	0.12
SZ	0.53	0.36
US	0.64	0.66

Notes: This table compares the weighted candidate novelty (WCN) for political parties running in the 1998 Chamber of Deputies election using two distinct datasets. The correlation between the two sources of estimates of the WCN is 0.95.

Table S3. Comparison CPCD and Party People dataset, Chamber of Deputies Election 2002
Weighted Candidate Novelty

Political Party	Sikk & Köker	CPCD
AZSD	1.00	1.00
BPS	1.00	1.00
CP	1.00	1.00
CSDH	0.45	0.80
CSNS	0.64	0.71
CSSD	0.37	0.31
CZ	0.87	0.75
DL	0.97	1.00
H.A.	1.00	1.00
KDU-CSL	0.22	0.16
KSCM	0.30	0.29
MoDS	0.61	0.60
N	0.96	0.94
NDS	0.91	0.92
NH	1.00	1.00
ODA	0.65	0.43
ODS	0.26	0.21
PB	0.91	0.91
REP	0.59	0.51
RMS	0.33	0.26
ROI	1.00	1.00
SDS	1.00	1.00
SNK	0.96	0.99
SV SOS	0.89	0.94
SZ	0.56	0.54
SZJ	0.60	0.48
SZR	1.00	1.00
VPB	1.00	1.00

Notes: This table compares the weighted candidate novelty (WCN) for political parties running in the 2002 Chamber of Deputies election using two distinct datasets. The correlation between the two sources of estimates of the WCN is 0.95.

Table S4. Comparison CPCD and Party People dataset, Chamber of Deputies Election 2006
Weighted Candidate Novelty

Political Party	Sikk & Köker	CPCD
4 VIZE	0.94	0.95
BPS	0.90	0.89
CHNJ	0.36	0.23
CSNS2006	0.31	0.36
CSSD	0.40	0.50
FiS	1.00	1.00
HOB	0.66	0.44
HS	0.28	0.30
KC	0.93	1.00
KDU-CSL	0.30	0.36
KSCM	0.12	0.18
Koal_CR	0.83	0.77
LiRA	1.00	1.00
M	0.51	0.40
NEZ	0.84	0.89
NEZ/DEM	0.70	0.81
NS	0.74	0.58
ODS	0.40	0.44
PB	0.48	0.44
PaS	0.61	0.48
SNK ED	0.45	0.54
SRS	0.96	0.95
SZ	0.69	0.64
SZR	0.87	0.88
US-DEU	0.62	0.68

Notes: This table compares the weighted candidate novelty (WCN) for political parties running in the 2006 Chamber of Deputies election using two distinct datasets. The correlation between the two sources of estimates of the WCN is 0.95.

Table S5. Comparison CPCD and Party People dataset, Chamber of Deputies Election 2010
Weighted Candidate Novelty

Political Party	Sikk & Köker	CPCD
CPS	0.96	1.00
CSNS	0.49	0.35
CSNS2005	0.56	0.18
CSSD	0.38	0.43
DSSS	0.58	0.54
ES	0.83	1.00
HS	0.27	0.30
KC	0.48	0.50
KDU-CSL	0.51	0.45
KH	1.00	1.00
KONS	0.88	0.86
KSCM	0.32	0.27
LIB	0.00	0.00
Moravane	0.65	0.80
ODS	0.46	0.49
Obcane	0.93	0.92
PB	0.45	0.26
SPOZ	0.90	0.87
SPR-RSC	0.47	0.70
STOP	0.97	1.00
SZ	0.54	0.59
Suveren.	0.82	0.82
Svobodni	0.91	0.98
TOP 09	0.73	0.79
VV	0.84	0.85

Notes: This table compares the weighted candidate novelty (WCN) for political parties running in the 2010 Chamber of Deputies election using two distinct datasets. The correlation between the two sources of estimates of the WCN is 0.92.

Table S6. **Comparison CPCD and Party People dataset, Chamber of Deputies Election 2013**
Weighted Candidate Novelty

Political Party	Sikk & Köker	CPCD
ANEO	1.00	1.00
ANO 2011	0.89	0.91
CSSD	0.38	0.39
DSSS	0.38	0.34
HLVZHURU	0.65	0.56
KAN	1.00	1.00
KC	0.62	0.63
KDU-CSL	0.46	0.37
KSCM	0.25	0.26
LEV 21	0.86	0.89
OBC_2011	1.00	1.00
ODS	0.54	0.61
PB	0.34	0.39
Pirati	0.49	0.48
RDS	1.00	1.00
SPOZ	0.77	0.75
SZ	0.46	0.63
SsCR	0.93	0.91
Suveren.	0.43	0.34
Svobodni	0.54	0.62
TOP 09	0.31	0.36
Usvit	0.80	0.81
Zmena	0.83	0.86

Notes: This table compares the weighted candidate novelty (WCN) for political parties running in the 2017 Chamber of Deputies election using two distinct datasets. The correlation between the two sources of estimates of the WCN is 0.97.

Supplementary Information C: Distribution of day-in-month donors' dates of birth

Figures S1, S2, and S3 present distribution of day-in-month donors' dates of birth.

Figure S1. Distribution of Day-in-Month Donors' Dates of Birth I

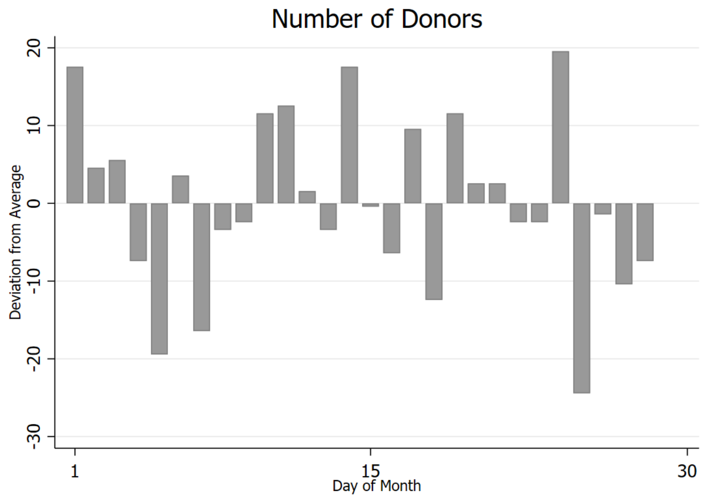

(a) KDU-ČSL

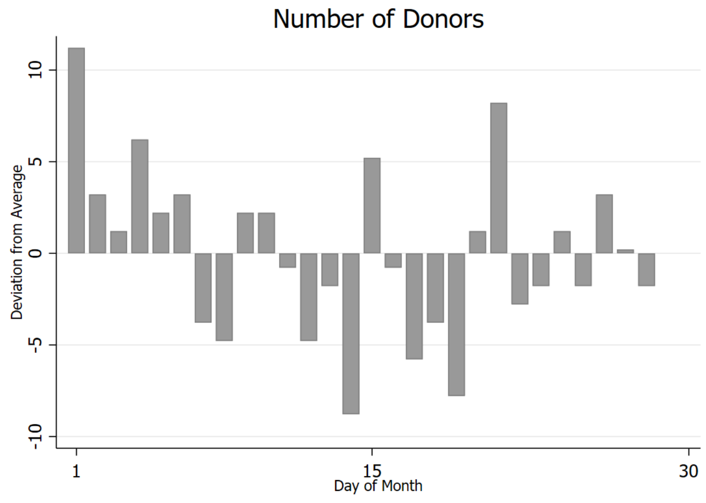

(b) KSČM

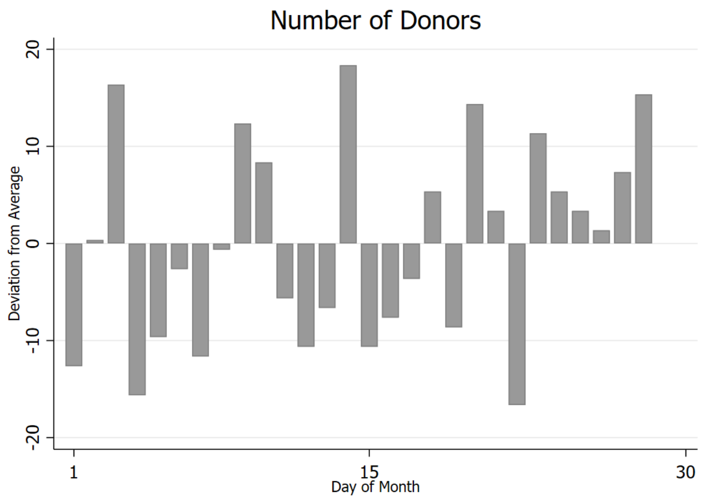

(c) ODS

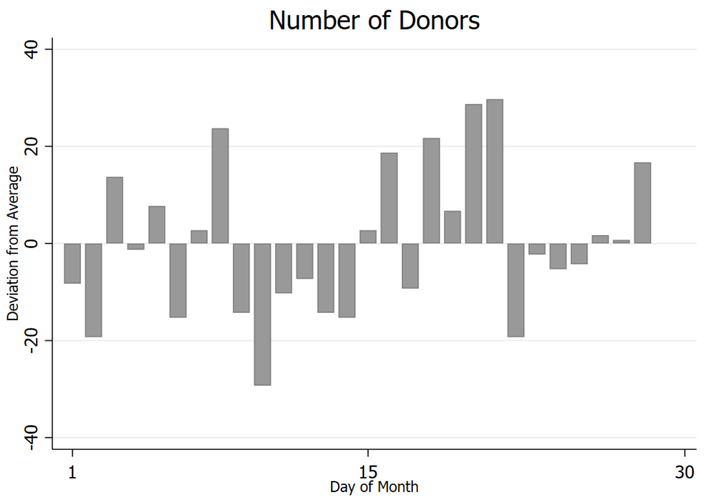

(d) Piráti

Figure S2. Distribution of Day-in-Month Donors' Dates of Birth II

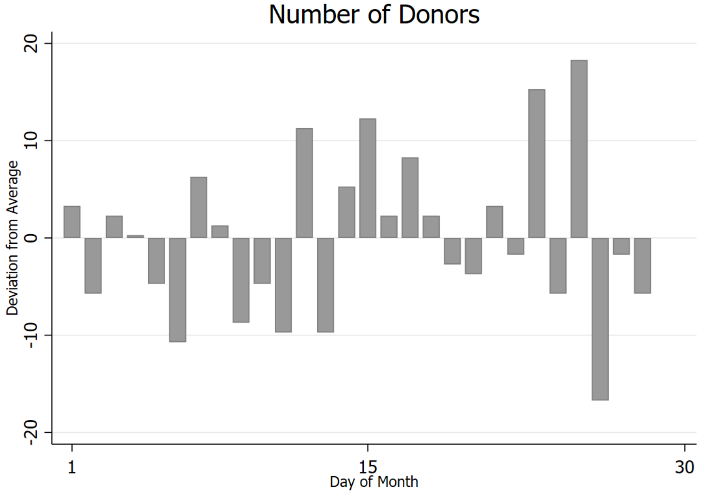

(a) SPD

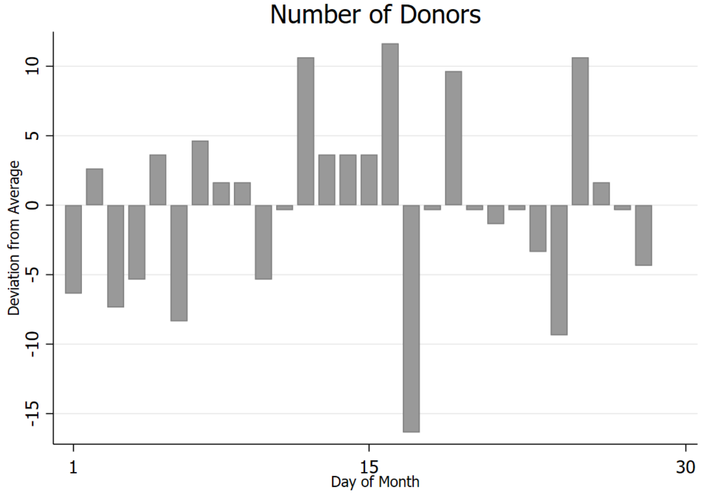

(b) STAN

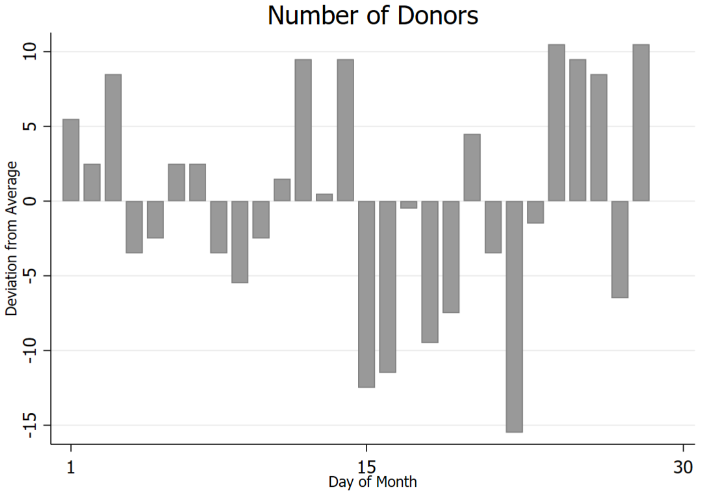

(c) TOP 09

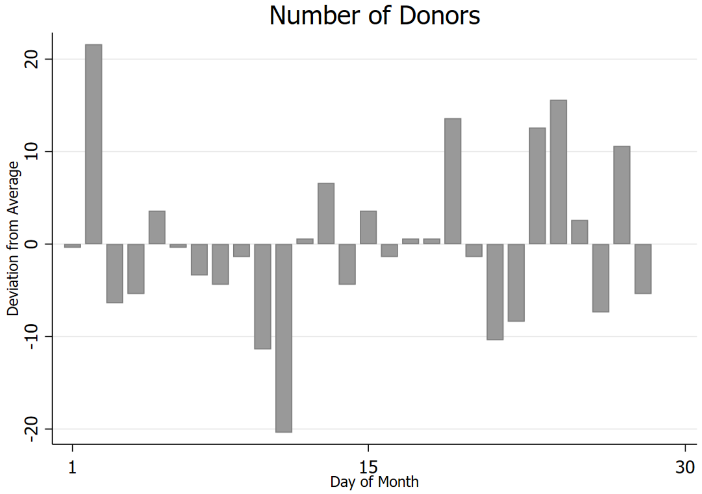

(d) ANO

Figure S3. Distribution of Day-in-Month Donors' Dates of Birth III

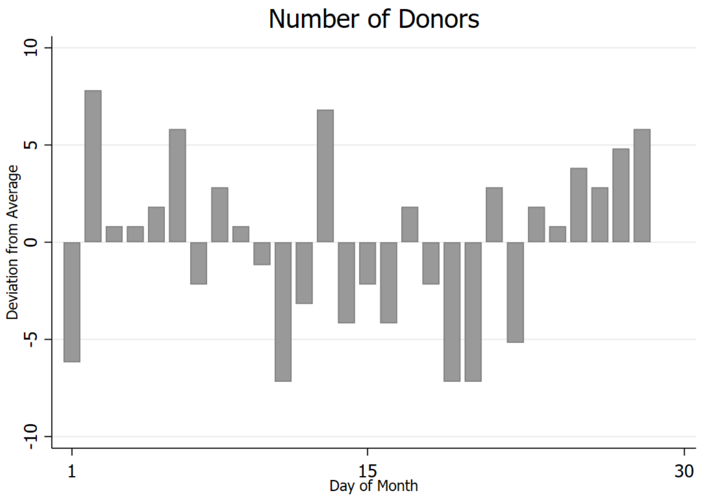

(a) ČSSD

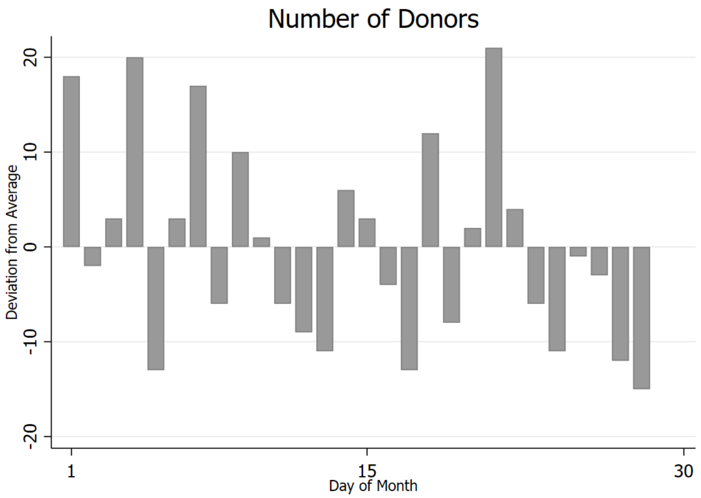

(b) Svobodní

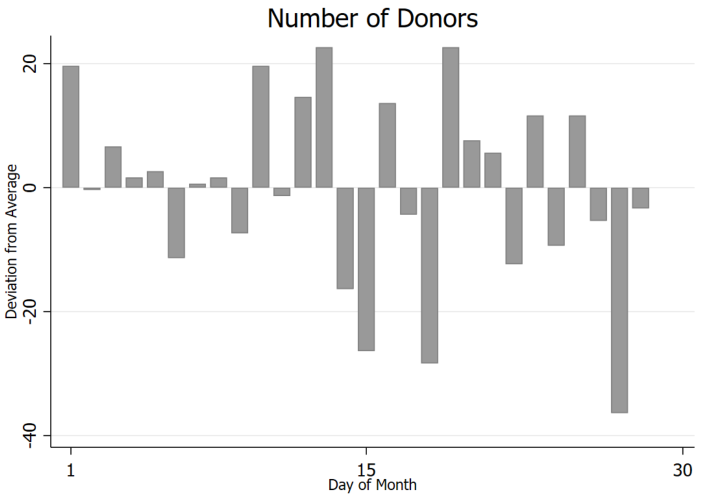

(c) Trikolora

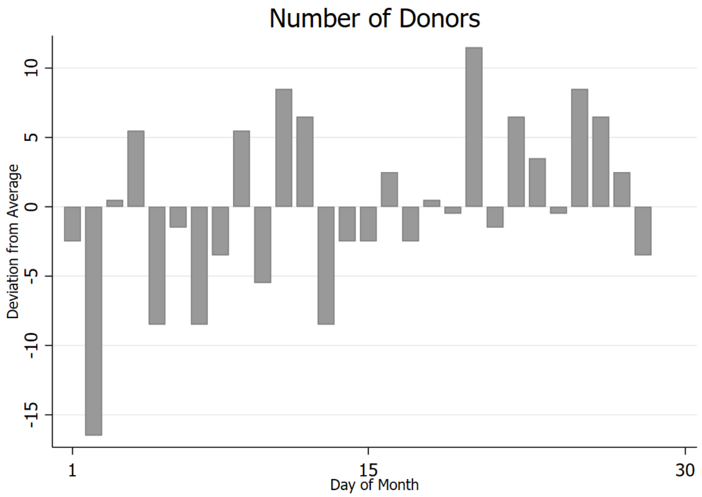

(d) Přísaha

References

1. Sikk, A. & Köker, P. *Party People: Candidates and Party Evolution* (Oxford University Press, Oxford, 2023).
